# Experimental and Numerical Study of the Hydrothermal Performance of Micro Pin Fin Heat Sinks with Streamwise-Varying Height

**DOI:** 10.3390/ma19040654

**Published:** 2026-02-08

**Authors:** Hang Gao, Dalei Jing

**Affiliations:** School of Mechanical Engineering, University of Shanghai for Science and Technology, Shanghai 200093, China; 18000132343@163.com

**Keywords:** micro pin fin heat sink, pin fin height, streamwise-varying height, hydrothermal performance

## Abstract

To enhance the hydrothermal performance of micro pin fin heat sinks (MPFHSs), this paper proposes five MPFHSs with different streamwise pin fin height variation modes and experimentally and numerically compares their hydrothermal performance, including pressure drop, maximum and average temperatures, and comprehensive performance evaluation criteria. The results indicate that, taking the uniform PFs height (UH) design as a reference, the designs with linearly increasing streamwise PFs height (LIH) and increasing streamwise PFs height with decelerating growth rate (DIH) demonstrate lower heat sink temperatures. Conversely, the designs with linearly decreasing streamwise PFs height (LDH) and decreasing streamwise PFs height with accelerating reduction rate (ADH) result in higher heat sink temperature. In addition, the comprehensive performance of LDH and ADH outperforms that of UH at low inlet flow rates, while the DIH surpasses that of UH at higher flow rates. As the inlet flow rate increases from 0.02 L/min to 0.5 L/min, our numerical study shows that the comprehensive performance of LDH and ADH decreases by 14.9% and 6.2%, respectively, whereas that of LIH and DIH increases by 17.4% and 10.2%, respectively. This finding provides insights to improve the hydrothermal performance of MPFHS.

## 1. Introduction

The relentless miniaturization and increasing power density of modern electronics, from high-performance computing chips to electric vehicle battery packs, have pushed thermal management to be a critical bottleneck limiting the performance, reliability, and lifespan of devices. The microchannel heat sink (MCHS) proposal by Pease and Tuckman [1] has been widely used for the thermal management of microelectronic chips [2,3], electric vehicle batteries [4,5], and photovoltaic energy storage systems [6,7], owing to its excellent heat dissipation efficiency and compact design. However, conventional MCHS designs face several challenges, such as significant temperature gradients and high energy consumption [8]. These challenges have driven extensive research efforts to improve the heat transfer and hydraulic performance of MCHS [9,10].

Among the various performance enhancement strategies, the integration of pin fins (PFs) into microchannels has gained considerable attention [11,12]. The high surface-area-to-volume ratio of PFs not only increases the solid–liquid interfacial area for heat transfer but also thins the thermal boundary layer, thereby improving heat transfer efficiency [13]. As a result, micro pin fin heat sinks (MPFHSs) have become a major focus of research in the field of thermal management techniques. Extensive optimization studies have been conducted for MPFHSs, focusing on how key parameters, including pin fin size, shape, and spatial distribution, affect both thermal and hydraulic performance [14,15].

Regarding the effects of PF size, Liu et al. [16] numerically investigated the effects of geometric parameters (diameter, length, and spacing) of circular needle fins on entropy production and the outlet temperature of nanofluids. The results indicate that increasing the length and diameter of the needle fins while optimizing their spacing can reduce both total entropy production and thermal entropy production while simultaneously lowering the outlet temperature of the nanofluid. Vasilev et al. [17] numerically and experimentally investigated the effects of circular PF size on the heat transfer performance of laminar flow inside an MCHS, reporting a 40% increase in Nusselt number at a PF height of 0.5 mm compared to a finless design. However, excessive PF height increases flow resistance, ultimately compromising thermal performance. Prajapati [18] numerically investigated the effect of PF height in a range of 0.4–1.0 mm on the heat transfer performance of an MCHS. They found that a PF height of 0.8 mm yields the best heat transfer performance. Bhandari et al. [19] numerically investigated the effect of square PF height in the range of 0.5 mm to 2.0 mm on the heat transfer and hydrodynamic performance of an open MCHS. They found that the square PFs with a height of 1.5 mm provided the best heat transfer performance.

The influences of the PF shapes on the heat transfer and hydraulic performance of MCHS have also been studied extensively. For example, Abdoli et al. [20] conducted numerical simulations to compare the effects of teardrop-shaped PFs and round PFs on the hydrothermal performance. They found that teardrop-shaped PFs outperformed round PFs with a 30.4% reduction in pump power, a 3.2% improvement in convection heat transfer ratio, and a 6.4 °C decrease in maximum temperature. Yang et al. [21] conducted combined numerical and experimental investigations on the performance of PFs with five distinct cross-sectional shapes, namely circular, triangular, square, pentagonal, and hexagonal. Their findings revealed that the geometric shape of PFs exerts a crucial influence on balancing flow pressure drop and heat transfer capacity: specifically, circular PFs yield the minimum pressure drop, while hexagonal cross-sectional PFs are conducive to enhancing heat transfer efficiency. Ambreen et al. [22] numerically analyzed the effects of square, circular, and hexagonal PFs on the heat transfer performance of nanofluid flow within the MCHSs. Their results exhibited that the circular PFs showed superior heat transfer performance, followed by hexagonal and square configurations. Alam et al. [23] numerically analyzed the heat transfer characteristics of triangular PF heat sinks with heights of 1.0 mm and 2.0 mm for different Reynolds numbers and inlet turbulence intensities. They found that the best heat transfer is achieved when the PF’s height is 2.0 mm and the inlet turbulence intensity is 20%.

In addition, the spatial distribution of the PFs can also enhance the thermal performance of the MCHS. Sallar et al. [24] numerically investigated the effects of height, thickness, and angle of I-shaped PFs on the heat transfer and hydraulic performance of MCHSs. They reported a 30% increase in overall performance compared to that of the structure without PFs by changing the PFs arrangement. Gao et al. [25] employed both experimental and numerical approaches to evaluate the hydrothermal performance of various PF arrangements and found that a gradient staggered configuration provides the optimal balance between heat transfer enhancement and flow resistance, with thermal performance reaching 4.8–6.1 times that of smooth channels. Şara [26] experimentally investigated the effect of staggered square PFs density on the heat transfer performance of MCHS and found that there is a positive correlation between PFs density and the heat transfer coefficient of the MCHS.

Despite this extensive body of work, most studies have focused exclusively on PFs of uniform height. A uniform PF array, however, presents a constant flow resistance and heat transfer area along the channel. This leads to an inefficient allocation of resources: the fins near the outlet, where the driving temperature difference is smaller, contribute less to heat transfer per unit of pumping power they incur. Considering the thermal performance is highly sensitive to PF height, especially at low Reynolds numbers [27,28], it is possible to tailor the flow resistance and heat transfer area to match the local thermal load by strategically varying the pin fin height in the streamwise direction.

Despite previous numerical studies by Li et al. [29] and Kumar et al. [30] that have suggested distinct advantages for variable-height configurations, the potential of variable-height PFs remains largely unexplored, and experimental validation is notably absent from the literature. To address this critical gap, this study proposes five distinct streamwise-varying PF height modes for an MPFHS. The hydrothermal performance of these configurations is systematically evaluated through an integrated approach of experimental analysis and numerical simulation. The findings aim to provide new insights and practical design guidelines for the next generation of high-performance microchannel heat sinks.

## 2. Experiments

### 2.1. Experimental Samples

**Figure 1:**
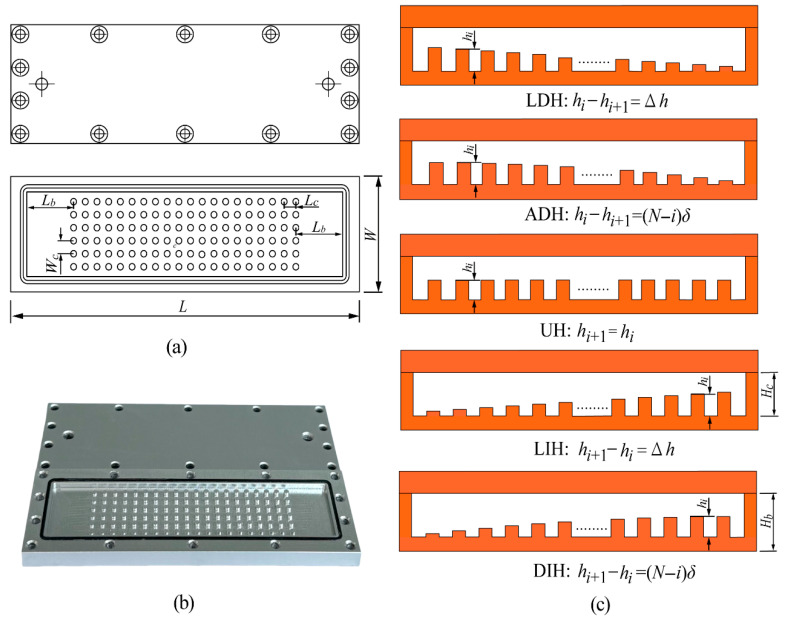
(**a**) Schematic of the sample, (**b**) photograph of the fabricated sample, and (**c**) five PFs height variation modes.

As illustrated in Figure 1, the experimental sample of heat sink with streamwise-varying height pin fin (HS-SVHPF) used in present work is a two-layer Aluminum 6061 structure fabricated using a high-speed computer numerical control engraving and milling machine (Shanghai Danmai Machinery Manufacturing Corporation, Shanghai, China). The upper layer is a 5 mm thick cover plate, and the lower layer is a base plate with a height of *H_b_* = 6 mm. The fluid channel with a height of *H_c_* = 2 mm is fabricated on the base plate, featuring circular PFs with a diameter of *d_f_* = 2 mm. The PFs are arranged in six evenly spaced rows with 20 linearly aligned PFs in each row. The transverse and longitudinal spacing distances between adjacent PFs are *W_c_* = 2 mm and *L_c_* = 2 mm. The distance between the outermost PFs and the sidewall is maintained at 2 mm. In addition, the distances from the PFs to the inlet and outlet are set to be *L_b_* = 16 mm to ensure a fully developed fluid flow of the coolant into the PFs section. The overall dimensions of the HS-SVHPF are *L* × *W* × *H* = 124 × 42 × 11 mm^3^.

To investigate the impact of PFs height variation on the hydrothermal performance of the HS-SVHPF, five distinct height variation modes are designed while maintaining a constant total PF volume, as depicted in Figure 1c. The five modes are described as follows:

**LDH:** PF height decreases linearly along the streamwise direction.

**ADH:** PF height decreases along the streamwise direction with an accelerating reduction rate.

**UH:** Uniform PF height throughout the channel (baseline configuration).

**LIH:** PF height increases linearly along the streamwise direction.

**DIH:** PF height increases along the streamwise direction with a decelerating growth rate.

The height values of the PFs for the five different modes are detailed in Table 1.

### 2.2. Experimental Setup

**Figure 2:**
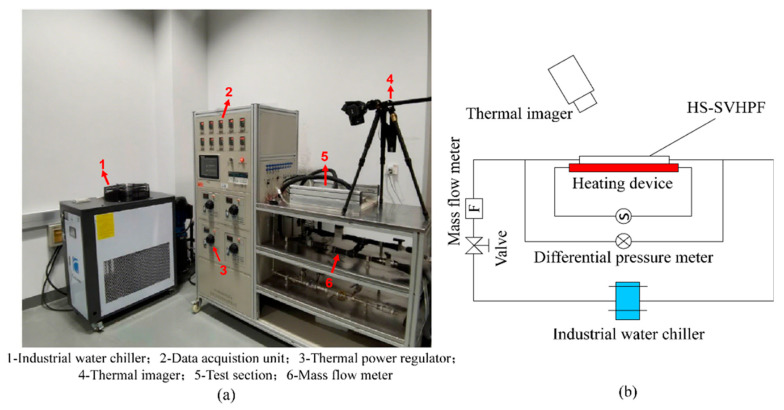
(**a**) Physical diagrams of the experimental system and (**b**) schematic.

Figure 2 illustrates the schematic and physical diagram of the experimental system used for assessing the hydrothermal performance of the HS-SVHPF. This experimental platform consists of three main components: a heating subsystem, a coolant circulation subsystem, and a measurement subsystem. In the heating subsystem, heat is transferred to the bottom surface of the HS-SVHPF via a red copper plate embedded with stainless-steel heating rods. A thermal power regulator (STG-3000W, Zhejiang Huizheng Electric Corporation, Wenzhou, China) is utilized to regulate the heating power, which is set to 200 W for this study. To ensure heat conduction between the heating subsystem and the HS-SVHPF and minimize thermal losses, a uniform layer of Qingmei-QM850 (Shenzhen Youdao New Materials Technology Corporation, Shenzhen, China) thermally conductive silver silicone grease is applied between the copper plate and the bottom surface of the HS-SVHPF. The coolant circulation subsystem employs an industrial water chiller (Coolsoon-CA02, Coolsoon Corporation, Shenzhen, China) to maintain a steady coolant inlet temperature of 20 °C. A combination of a control valve and a mass flow meter (Omega-flv 4605A, DwyerOmega Corporation, Michigan City, IN, USA) within the recirculation pipeline allows for precise regulation of the coolant flow rate. The flow rates selected for the experiments are 0.1, 0.2, and 0.3 L/min. A relatively long connecting tube (approximately 30 cm in length) is installed before both the inlet and outlet of the heat sink. This long, white, flexible tube is used as the inlet section to ensure the fluid attains fully developed flow.

To evaluate the thermal performance of the HS-SVHPF, the upper surface temperature of the heat sink was recorded using an infrared thermal imager (FLIR T630SC, FLIR Systems Corporation, Wilsonville, OR, USA). This camera has a resolution of 640 × 480 pixels, a temperature measurement range of −40 °C to 650 °C, a spectral range of 7.5–13 μm, and a specified accuracy of ±2 °C. It produces pseudo-color thermal images for quantitative temperature analysis. During the experiments, the FLIR T630SC was used with a fixed emissivity and a temperature span set to 20–60 °C. To mitigate the influence of surface emissivity variations, the sample was coated with silver silicone grease. The camera’s automatic emissivity calibration function was also employed to enhance measurement accuracy. The thermal imager was vertically suspended 0.5 m directly above the heat sink, with its lens oriented at a 90° viewing angle relative to the surface. This setup minimized potential obliquity errors. The reflected ambient temperature parameter was set to the laboratory environment temperature (25 °C) to correct for background radiation interference. An uncertainty analysis determined that the combined temperature measurement uncertainty from emissivity variations and reflection errors was within ±1 °C, which is consistent with the camera’s specified accuracy. A differential pressure meter (Meacon MIK3051, Meacon Corporation, Hangzhou, China) was employed to measure the total pressure drop across the HS-SVHPF system. Pressure sampling points were positioned on the connecting tubes located immediately before the heat sink inlet and after its outlet. Consequently, the measured pressure drop includes both the loss across the heat sink itself and the frictional losses within these connecting tubes. Data were recorded at 10 s intervals, with 10 data points collected per sampling set. A total of 5 independent tests were conducted, and the average value derived from multiple measurements was adopted as the characteristic parameter for evaluating the hydraulic performance of the system. The average of these values was then used to characterize the hydraulic performance of the system. To enhance experimental accuracy, the coolant circulation pipeline is insulated with foam wool to mitigate heat dissipation during the tests.

### 2.3. Heat Loss and Uncertainty Analysis

To assure the accuracy of the experiments, the heat loss analysis and the uncertainty analysis are performed as follows. The heat loss to the ambient is estimated by the following [31]:

(1) $$Heat\;loss=\frac{Q_{electr}-Q_{coolant}}{Q_{electr}}$$

where $Q_{electr}$ is the transformer output power, $Q_{coolant}$ is the heat power gained by cooling water, and m denotes the mass flow rate. In this study, the average heat loss is calculated to be less than 5%.

The uncertainty analysis of the heat flux gained by coolant water is performed using the root square (RSS) method [31,32]:

(2) $$u_{R,RSS}=\sqrt{\sum_{i=1}^{N}(u_{i}\frac{\partial R}{\partial x_{i}})}$$

where $R=R(x_{1},x_{2},x_{3},\ldots ,x_{n})$ is a functional relationship of independent variables $x_{n}$. $u_{R,RSS}$ is the uncertainty of *R*. The heat flux gained by coolant water $q_{coolant}$ can be calculated by the following:

(3) $$q_{coolant}=\frac{Q_{coolant}}{A}=\frac{c_{p}m∆T}{L\times W}$$

where A is the heating area. Then, the synthetic uncertainty of $q_{coolant}$ can be calculated as follows:

(4) $$u_{R}=\sqrt{{\left[u_{m}(\frac{\partial q_{coolant}}{\partial m})\right]}^{2}+{\left[u_{∆T}(\frac{\partial q_{coolant}}{\partial ∆T})\right]}^{2}+{\left[u_{L}(\frac{\partial q_{coolant}}{\partial L})\right]}^{2}+{\left[u_{W}(\frac{\partial q_{coolant}}{\partial W})\right]}^{2}}$$

The uncertainty calculated from the RSS method for the heat flux gained by coolant is less than 6%.

## 3. Numerical Setup

### 3.1. Numerical Method

In addition to experimental investigations, the hydrothermal performance of the HS-SVHPFs is also analyzed numerically. To simplify the simulation, the following assumptions are adopted,

(1) Three-dimensional steady-state single-phase laminar flow and heat transfer.
(2) The liquid is an incompressible Newtonian fluid.
(3) The natural convection heat transfer is set between the outer surfaces of the HS-SVHPF and the surrounding environment to simulate the experimental process.
(4) Physical properties of solids and fluids do not change with temperature.
(5) Neglecting the effects of viscous dissipation and gravity.

Based on the above assumptions, the governing equations for the HS-SVHPF are given below [29]:

Mass conservation equation in the fluid domain:

(5) $$\nabla \cdot \vec{U_{l}}=0$$

where $\vec{U_{l}}$ denotes the velocity field.

Momentum conservation equation for the fluid:

(6) $$\rho (\vec{U_{l}}\cdot \nabla \vec{U_{l}})=-\nabla P+\nabla \cdot (\mu_{l}\nabla \vec{U_{l}})$$

where P represents the pressure field, ρ is the density, and μ is the viscosity.

Energy conservation equation for fluid flow:

(7) $$\rho C_{pl}\left(\vec{U_{l}}\cdot \nabla T_{l}\right)=k_{l}\nabla^{2}T_{l}+q$$

Energy conservation equation for a solid:

(8) $$k_{s}\nabla^{2}T_{s}=0$$

where T is the temperature field, k and $C_{p}$ are the thermal conductivity and specific heat capacity. The subscript *l* and s denotes the liquid and solid. $Q_{electr}$ is the heat source on the bottom of the HS-SVHPF, equaling to the sum of $Q_{coolant}$ and the natural thermal convection $Q_{b}$ between the external environment and the outer surface of the HS-SVHPF, which can be calculated by the following: [33]

(9) $$Q_{electr}=Q_{coolant}+Q_{b}$$

(10) $$Q_{b}=h_{f}(T_{ext}-T_{sur})$$

where $T_{ext}$ is the external ambient temperature, $T_{sur}$ is the surface temperature of the HS-SVHPF, and $h_{f}$ represents the natural convective heat transfer coefficient, which depends on the ambient flow conditions and the surface geometry of the HS-SVHPF.

Based on the experimental setup, the initial and boundary conditions employed in the numerical analysis are listed as follows.

(1) Inlet: Fully developed velocity profile with a temperature of 20 °C. The corresponding flow rate selected for simulation is in the range of 0.02–0.5 L/min.
(2) Outlet: The outlet pressure is set at zero.
(3) Solid–liquid interface: No slip, no heat accumulation or loss at the interface, and heat flow is continuous through the interface.
(4) Thermal boundary: The bottom of the heat sink is set with constant heating power $Q_{electr}$ = 200 W to keep consistency with the experimental setup, and the side and top surfaces of the heat sink are set to be external natural convection boundary conditions.

In this study, the material for the heat sink is selected as aluminum, and the coolant is selected as water. The physical properties of aluminum and water are listed in Table 2. The computational domain is primarily meshed with free hexahedral elements, while angular refinement is performed on sharp corners, edges, and regions with large curvature of the fluid domain geometric model. Meanwhile, the boundary layer mesh is refined with 2 boundary layers, a stretching factor of 1.2, and a thickness adjustment factor of 5. To ensure good convergence, the successive over-relaxation (SOR) iterative method is adopted to solve the linear system of equations. In the iterative calculation, convergence is deemed achieved when the maximum residual was less than 10^−7^. Based on the aforementioned numerical settings, the steady-state solver of the multiphysics software COMSOL 6.1 is employed to solve the current problem.

### 3.2. Mesh Convergence Test

To ensure the accuracy of the numerical analysis, a grid independence study is first conducted using the UH configuration at an inlet flow rate of *V*_in_ = 0.3 L/min. Four hexahedral meshes with different grid numbers were used for the simulation calculations. The results for the pressure loss ∆P and the average bottom surface temperature $T_{b}$ are summarized in Table 3. Compared to the secondary mesh, the third mesh configuration achieves an optimal balance between computational efficiency and accuracy, with the change in ∆P less than 2% and the variation in $T_{b}$ less than 1%. Consequently, this mesh configuration is adopted for all subsequent simulations.

### 3.3. Performance Index

The following performance parameters are introduced to characterize the hydrothermal performance and overall performance of the HS-SVHPF.

(1) Pressure drop ∆P is used to characterize hydraulic performance.

(11) $$∆P=P_{in}-P_{out}$$

where $P_{in}$ and $P_{out}$ are the average pressure at the inlet and outlet of the sample connecting tubes.

(2) The temperature properties of HS-SVHPF are characterized in terms of the average and maximum temperatures on the top surface of the heat sink, $T_{us\_avg}$ and $T_{us\_max}$, the overall maximum temperature at the solid–liquid contact area of the heat sink, $T_{hs\_max}$, and the average temperature $T_{bs\_avg}$ on the heated bottom surface of the heat sink, where, $T_{us\_avg}$ and $T_{us\_max}$ include both experimental and numerical results, while $T_{hs\_max}$ and $T_{bs\_avg}$ are numerical results.
(3) Comprehensive performance evaluation criterion η, which is a comprehensive performance parameter used to characterize overall hydraulic and thermal performance [29,34].

(12) $$\eta =(\frac{Nu}{{Nu}_{ref}})/{\left(\frac{f}{f_{ref}}\right)}^{1/3}$$

where ${Nu}_{ref}$ and $f_{ref}$ are the Nusselt number and friction coefficient of the heat sink with uniform PFs height at the same flow rate. f is the friction coefficient and can be calculated as follows [35]:

(13) $$f=\frac{2∆P}{\left(\frac{L}{D_{h}}\right)\rho u_{in}^{2}}$$

where L is the heat sink length, $D_{h}$ is the hydraulic diameter of the mini-channel inlet, and $u_{in}$ = $V_{in}/A_{c}$ is the fluid inlet velocity. $D_{h}$ is given as follows:

(14) $$D_{h}=\frac{4A_{c}}{L_{c}}$$

where $A_{c}$ is the cross-sectional area of the flow channel in the yz direction and $L_{c}$ is the perimeter of the entrance.

Nusselt number is given as follows [29]:

(15) $$Nu=\frac{hD_{h}}{k_{l}}$$

where h is the convective heat transfer coefficient given as follows [29]:

(16) $$h=\frac{Q_{coolant}}{S_{w}(T_{w}-T_{f})}$$

where $Q_{coolant}=C_{p}m(T_{out}-T_{in})$ is the quantity of heat transfer [36], $S_{w}$ is solid–liquid contact area, and $T_{w}$ is the average temperature of solid–liquid contact interface and $T_{f}$ is the average fluid temperature, $C_{p}$, and $k_{l}$ parameters for coolant at 20 °C.

## 4. Results and Discussion

### 4.1. The Hydraulic Performance

**Figure 3:**
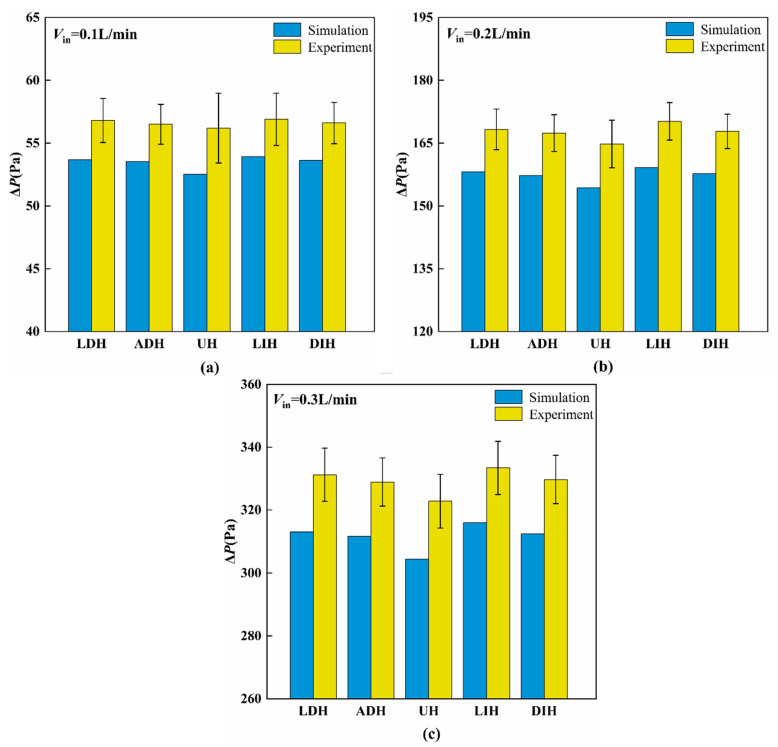
Comparisons of numerical and experimental Δ*P* under different inlet flow rates: (**a**) *V*_in_ = 0.1 L/min, (**b**) *V*_in_ = 0.2 L/min, and (**c**) *V*_in_ = 0.3 L/min.

Figure 3 illustrates the pressure drop (∆*P*) resulting from both simulations and experiments for the HS-SVHPF with different PF height variation modes at inlet flow rates of *V*_in_ = 0.1 L/min, 0.2 L/min, and 0.3 L/min. The numerical and experimental results exhibit good agreement with each other. Their errors are attributed to pressure losses in the connecting pipelines between the inlet and outlet of the heat sink to the differential pressure meter, as well as flow rate fluctuations in the coolant circulation system. The results in Figure 3 indicate that the ∆*P* increases with the increasing *V*_in_ for all five HS-SVHPFs. This trend results from the enhanced flow momentum proportionally to velocity, which leads to greater viscous losses along the channel.

**Figure 4:**
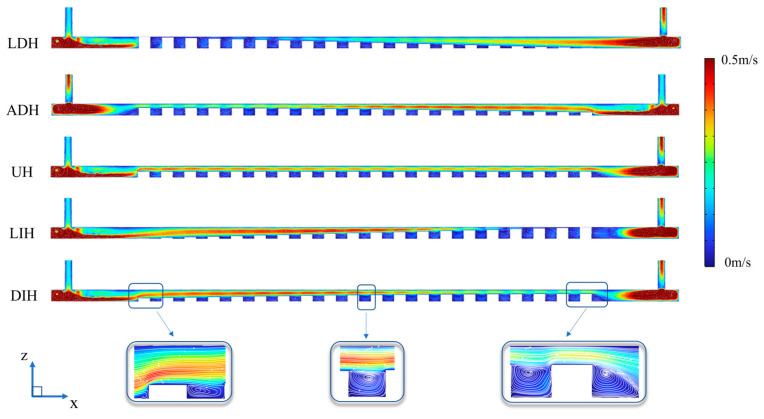
Velocity fields at the *y* = 19 mm plane of the five HS-SVHPFs when *V*_in_ = 0.3 L/min.

Among the various PF height variation modes, the HS-SVHPF with UH design demonstrates the lowest pressure drop. This phenomenon can be explained by analyzing cross-sectional area variations throughout the channel. In contrast to the UH design, the other four HS-SVHPF designs featuring streamwise-varying PFs height change the cross-sectional height for liquid transportation. For the LDH and ADH designs, where PFs’ height decreases along the flow direction, the upstream cross-sectional height is constricted while the downstream height expands. This creates greater upstream flow blockage and reduced downstream blockage. Conversely, the LIH and DIH designs, characterized by increasing PFs height in the flow direction, exhibit expanded upstream cross-sectional height and constricted downstream height, leading to diminished upstream blockage and enhanced downstream blockage. This can be further verified by the velocity distribution with the HS-SVHPFs, as given in Figure 4. The UH configuration, characterized by a constant PFs height and a uniform cross-sectional area, achieves the minimum pressure loss. This is attributed to the fact that a smaller flow channel induces a higher pressure drop. In contrast, the other four configurations exhibit significant channel contraction at either the inlet or outlet, resulting in substantial pressure loss and consequently a higher pressure drop compared to the UH configuration.

### 4.2. The Thermal Performances

**Figure 5:**
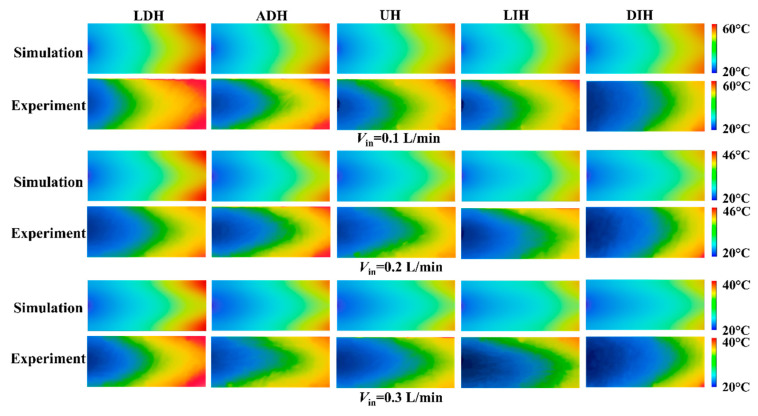
The numerical and experimental temperature contours on the top surface of the HS-SVHPFs with different PF height variation modes at three inlet flow rates.

To analyze the effects of five PF height variation modes on the thermal performance of the HS-SVHPFs, Figure 5 first gives the numerical and experimental temperature contours on the top surface of the five HS-SVHPFs at the inlet flow rates of *V*_in_ = 0.1 L/min, 0.2 L/min, and 0.3 L/min. The numerical and experimental results are in good consistency with each other, indicating the reliability of the numerical simulation and experiment. From the temperature contours, it is evident that the top surface temperatures of the five HS-SVHPFs decrease with the increasing inlet flow rates. This is attributed to the thinning thermal boundary layer caused by the increasing flow rate, which shortens the heat diffusion distance from the wall to the fluid. Furthermore, the different PF height variation modes exhibit heat transfer performance. The streamwise-decreasing PFs height design exhibits larger temperature gradients, but the streamwise-increasing PFs height design exhibits smaller temperature gradients in comparison to the UH design.

**Figure 6:**
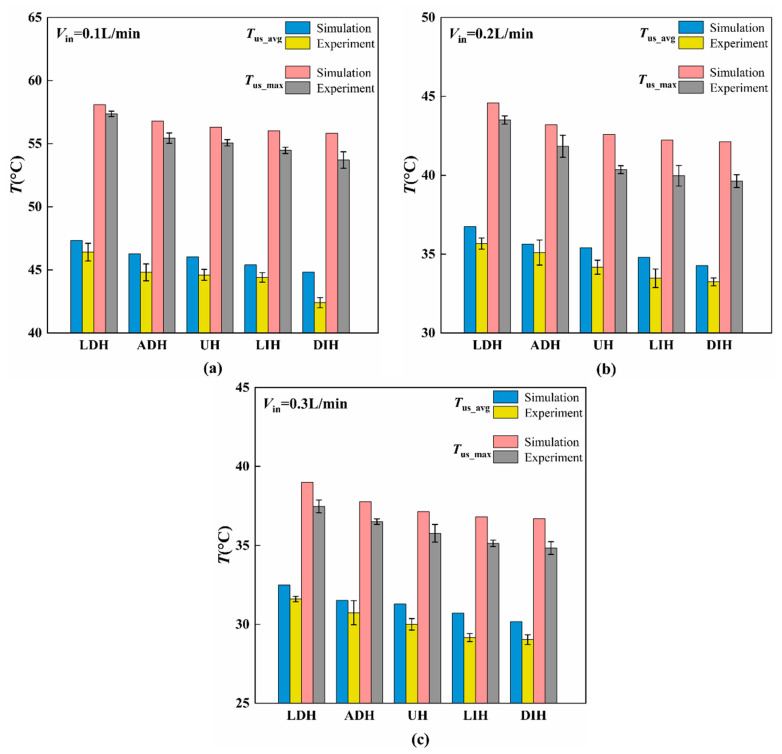
Comparisons of numerical and experimental top surface temperature at different inlet flow rates of (**a**) *V*_in_ = 0.1 L/min, (**b**) *V*_in_ = 0.2 L/min, and (**c**) *V*_in_ = 0.3 L/min.

In order to quantitatively analyze the influence of PFs height variation modes on the heat transfer performance, Figure 6 gives the experimental and numerical average temperatures, *T*_us_avg_, and maximum temperatures, *T*_us_max_, on the top surface of the HS-SVHPFs. From the results given in Figure 6, it can be found that compared to the UH design, the two streamwise height-decreasing modes, LDH and ADH, exhibit higher top surface temperatures; however, the two streamwise height-increasing modes, LIH and DIH, exhibit lower top surface temperatures. Among them, the *T*_us_avg_ and *T*_us_max_ of the LDH configuration are the largest compared to the other four modes, which indicates the thermal performance of LDH is the worst, while the *T*_us_avg_ and *T*_us_max_ of DIH are the smallest among the five configurations, indicating the best thermal performance. In detail, the average top surface temperature *T*_us_avg_ and maximum top surface temperature *T*_us_max_ of DIH decreased 8.6% and 6.8%, 7.3% and 8.8%, and 8.9% and 8.2%, respectively, compared to the LDH when the inlet volume flow rates were *V*_in_ = 0.1, 0.2, and 0.3 L/min based on the experimental results. Additionally, when the volume of *V*_in_ is substantial, the LIH and DIH designs can significantly enhance secondary flow by increasing the height of the downstream PFs. This enhancement, coupled with elevated flow velocity, reduces the thickness of the thermal boundary layer and improves heat transfer performance. However, the increase in the height of downstream PFs also results in greater form drag and contraction loss, which consequently leads to an increase in Δ*P*.

**Figure 7:**
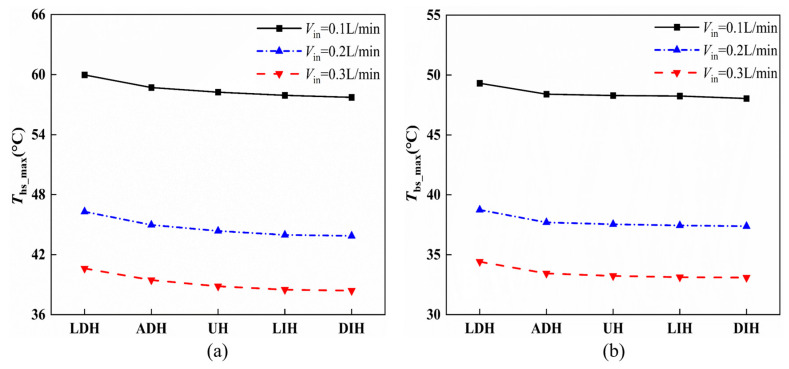
The effects of different *V*_in_ on the (**a**) numerical maximum heat sink temperature and (**b**) numerical average temperature on the bottom surface.

Besides the top surface temperatures, Figure 7 gives the maximum heat sink temperature, *T*_hs_max_, and the average temperature, *T*_bs_avg_, on the heated bottom surface based on the numerical simulation. It can be seen from Figure 7 that *T*_hs_max_ and *T*_bs_avg_ of all five HS-SVHPFs show similar trends with the maximum and average top surface temperatures. When *V*_in_ = 0.1, 0.2, and 0.3 L/min, *T*_hs_max_ of DIH is decreased by 2.25 °C, 2.42 °C, and 2.23 °C, respectively, compared to the *T*_hs_max_ of LDH. *T*_bs_avg_ of DIH is decreased by 1.27 °C, 1.36 °C, and 1.32 °C, respectively, compared to the *T*_bs_avg_ of LDH. For the two streamwise increasing PF height designs, the DIH design exhibits a lower temperature than that of the LIH design. This is because for the LIH configuration, the PF’s height at the downstream of the channel is excessively high, nearly coinciding with the upper wall of the heat sink. This restricts the flow of coolants and compresses the space for convective heat transfer in this region, resulting in a decrease in the heat transfer rate. This result aligns with the previous studies showing that excessively high uniform PFs also reduce the heat transfer for the uniform configuration [18,30].

### 4.3. Comprehensive Performance

**Figure 8:**
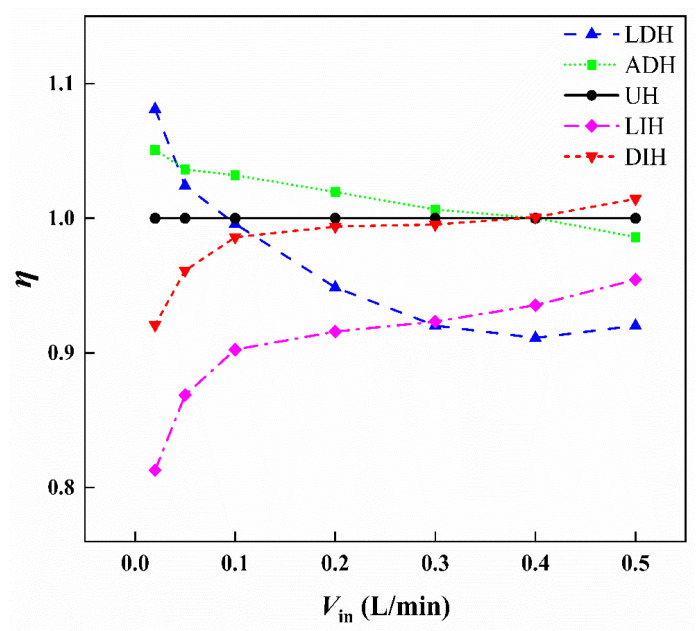
The η of the five HS-SVHPFs under different *V*_in_ based on numerical simulation.

From the above hydraulic and thermal performance analysis, it is evident that the HS-SVHPFs with reduced temperature are usually accompanied by increased pressure loss. Thus, it is necessary to assess the combined hydrodynamic and heat transfer performance. To better illustrate the trend of the comprehensive performance evaluation criterion η, Figure 8 gives the variation in the comprehensive performance evaluation criterion η of five HS-SVHPFs as the inlet flow rate increases in a wide range from 0.05 L/min to 0.5 L/min based on the numerical analysis. The UH design is selected as the reference. From Figure 8, the comprehensive performance evaluation criterion η of LDH and ADH designs, whose PFs height decreases along the streamwise direction, decreases with the increase in inlet flow rate, whereas the η of LIH and DIH designs, whose PFs height increases along the streamwise direction, increase with the increase in inlet flow rate. As the inlet flow rate increases from 0.02 L/min to 0.5 L/min, the comprehensive performance of LDH and ADH decreased by 14.9% and 6.2%, respectively, but the comprehensive performance of LIH and DIH increased by 17.4% and 10.2%, respectively.

Based on the definition of η, the comprehensive performance is proportional to *Nu* but inversely proportional to *f*^1/3^. For all the designs studied in the present work, both *Nu* and *f* increase as the flow rate increases. This leads to a trade-off between hydraulic performance and thermal performance. Consequently, the comprehensive performance may either improve or deteriorate, depending on the relative changes in *Nu* and *f*^1/3^. Compared to the UH design, at a small inlet flow rate, the LDH and ADH designs exhibit better comprehensive performance, because when the flow rate is very low, both the maximum and average temperatures on the top surfaces of LDH and ADH are higher than those of UH. However, the temperature difference ($T_{w}$ − $T_{f}$) for LDH and ADH is smaller compared to UH, resulting in higher Nu values for LDH and ADH than for UH. However, the DIH design exhibits better comprehensive performance than that of the UH design at the large inlet flow rate.

## 5. Conclusions

In this paper, the effects of five PF arrays with varying streamwise heights on the hydrothermal performance of MPFHS at different flow rates are investigated numerically and experimentally. The conclusions are summarized as follows.

(1) The pressure drops for all five HS-SVHPFs increase with increasing inlet flow rate, while the pressure drop of the UH design is the smallest, which suggests that either increasing or decreasing the PFs height along the streamwise direction increases the pressure loss of the HS-SVHPF.
(2) Among the five HS-SVHPFs, the LIH and DIH designs with increasing streamwise PFs height exhibit lower temperatures compared to the UH design, which indicates that the increasing PFs height along the streamwise direction is beneficial to reduce the temperature gradient.
(3) In terms of comprehensive performance, the LDH and ADH designs with decreasing PFs height along the streamwise direction show a decrease in performance with rising inlet flow rate. Conversely, the LIH and DIH designs with increasing PFs height along the streamwise direction demonstrate improved performance as the inlet flow rate increases. Compared to the UH design, the LDH and ADH designs exhibit superior comprehensive performance at lower flow rates. The DIH design achieves the best comprehensive performance at a higher inlet flow rate.

This study provides deeper insights into the performance characteristics of micro pin fin heat sinks and offers valuable guidance for their performance optimization. To further advance this field, future research should concentrate on the coupled optimization of PF height distributions along both the spanwise and streamwise directions. Such a comprehensive approach could facilitate additional improvements in the hydrothermal performance.

## Figures and Tables

**Table 1:** PFs Height within the HS-SVHPFs with different height variation modes (unit: mm, tolerance for sample fabrication is ±0.004 mm).

	LDH	ADH	UH	LIH	DIH
*h*_1_	1.95	1.266	1	0.05	0.506
*h*_2_	1.85	1.262	1	0.15	0.582
*h*_3_	1.75	1.254	1	0.25	0.654
*h*_4_	1.65	1.242	1	0.35	0.722
*h*_5_	1.55	1.226	1	0.45	0.786
*h*_6_	1.45	1.206	1	0.55	0.846
*h*_7_	1.35	1.182	1	0.65	0.902
*h*_8_	1.25	1.154	1	0.75	0.954
*h*_9_	1.15	1.122	1	0.85	1.002
*h*_10_	1.05	1.086	1	0.95	1.046
*h*_11_	0.95	1.046	1	1.05	1.086
*h*_12_	0.85	1.002	1	1.15	1.122
*h*_13_	0.75	0.954	1	1.25	1.154
*h*_14_	0.65	0.902	1	1.35	1.182
*h*_15_	0.55	0.846	1	1.45	1.206
*h*_16_	0.45	0.786	1	1.55	1.226
*h*_17_	0.35	0.722	1	1.65	1.242
*h*_18_	0.25	0.654	1	1.75	1.254
*h*_19_	0.15	0.582	1	1.85	1.262
*h*_20_	0.05	0.506	1	1.95	1.266

**Table 2:** The physical properties of water and aluminum [33].

Properties	Symbol	Water	Aluminum	Unit
Specific heat	*C_p_*	4183	900	J/(kg·K)
Thermal conductivity	*k*	0.599	238	W/(m·K)
Density	*ρ*	998.2	2700	kg/m^3^
Dynamic viscosity	*μ*	0.001004	\	Pa·s

**Table 3:** The mesh sensitivity test.

Test *i*	Grid Number	∆P (Pa)	$\frac{\left|{∆P}^{i+1}-{∆P}^{i}\right|}{{∆P}^{i}}$ (%)	$T_{b}$ (°C)	$\frac{\left|{T_{b}}^{i+1}-{T_{b}}^{i}\right|}{{T_{b}}^{i}}$ (%)	Computing Time
1	540,491	241.97		30.54		10 min 18 s
2	5,729,942	212.09	12.35	32.99	7.42	50 min 32 s
3	10,503,059	215.58	1.65	33.24	0.75	1 h 52 min 12 s
4	15,369,901	219.10	1.63	33.43	0.57	3 h 35 min 58 s

## Data Availability

The original contributions presented in this study are included in the article. Further inquiries can be directed to the corresponding author.
